# Time trends in real-world treatment patterns and survival in patients diagnosed with de novo HER2+ metastatic breast cancer: an analysis of the SONABRE registry

**DOI:** 10.1007/s10549-023-07235-0

**Published:** 2024-02-21

**Authors:** Sandra M. E. Geurts, Khava I. E. Ibragimova, Nan Ding, Marissa Meegdes, Frans Erdkamp, Joan B. Heijns, Jolien Tol, Birgit E. P. J. Vriens, Marcus W. Dercksen, Kirsten N. A. Aaldering, Manon J. A. E. Pepels, Linda van de Winkel, Natascha A. J. B. Peters, Agnes J. van de Wouw, Sabrina A. J. G. Maaskant, Nathalie J. A. Teeuwen-Dedroog, Thiemo J. A. van Nijnatten, Maaike de Boer, Vivianne C. G. Tjan-Heijnen

**Affiliations:** 1 Department of Medical Oncology, GROW– School for Oncology and Reproduction, Maastricht University Medical Center, PO BOX 5800, 6202 AZ Maastricht, The Netherlands; 2 Department of Internal Medicine, Zuyderland Medical Center, Sittard-Geleen, The Netherlands; 3 Department of Medical Oncology, Amphia, Breda, The Netherlands; 4 Department of Medical Oncology, Jeroen Bosch Hospital, Den Bosch, The Netherlands; 5 Department of Internal Medicine, Catharina Hospital, Eindhoven, The Netherlands; 6 Department of Medical Oncology, Máxima Medical Center, Eindhoven, The Netherlands; 7 Department of Internal Medicine, Laurentius Hospital, Roermond, The Netherlands; 8 Department of Internal Medicine, Elkerliek Hospital, Helmond, The Netherlands; 9 Department of Internal Medicine, St Anna Hospital, Geldrop, The Netherlands; 10 Department of Internal Medicine, Sint Jans Gasthuis Hospital, Weert, Netherlands; 11 Department of Internal Medicine, Viecuri Medical Centre, Venlo, The Netherlands; 12 Department of Surgery, Máxima Medical Center, Eindhoven, The Netherlands; 13 Department of Radiology and Nuclear Medicine, GROW, Maastricht University Medical Center, Maastricht, The Netherlands

**Keywords:** Breast neoplasms, ERBB2 protein, human, Neoplasm metastasis, Pertuzumab, Registries, Survival

## Abstract

**Purpose:**

The aim was to determine whether the real-world first-line progression-free survival (PFS) of patients diagnosed with de novo human epidermal growth factor receptor 2 positive (HER2+) advanced breast cancer (ABC) has improved since the introduction of pertuzumab in 2013. In addition to PFS, we aimed to determine differences in overall survival (OS) and the use of systemic and locoregional therapies.

**Methods:**

Included were patients systemically treated for de novo HER2+ ABC in ten hospitals in 2008–2017 from the SONABRE Registry (NCT-03577197). First-line PFS and OS in 2013–2017 versus 2008–2012 was determined using Kaplan–Meier analyses and multivariable Cox proportional hazards modelling. First-given systemic therapy and the use of locoregional therapy within the first year following diagnosis were determined per period of diagnosis.

**Results:**

Median and five-year PFS were 26.6 months and 24% in 2013–2017 (*n* = 85) versus 14.5 months and 10% in 2008–2012 (*n* = 81) (adjusted *HR* = 0.65, 95%*CI*:0.45–0.94). Median and five-year OS were 61.2 months and 51% in 2013–2017 versus 26.1 months and 28% in 2008–2012 (adjusted *HR* = 0.55, 95%*CI*:0.37–0.81). Of patients diagnosed in 2013–2017 versus 2008–2012, 84% versus 60% received HER2-targeted therapy and 59% versus 0% pertuzumab-based therapy as first-given therapy. Respectively, 27% and 23% of patients underwent locoregional breast surgery, and 6% and 7% surgery of a metastatic site during the first year following diagnosis.

**Conclusion:**

The prognosis of patients with de novo HER2 + ABC has improved considerably. Since 2013 one in four patients were alive and free from progression on first-given therapy for at least five years.

## Introduction

In high-income countries, around five percent of patients with a primary breast cancer diagnosis have metastatic disease at time of the primary diagnosis (i.e. de novo metastatic) [1]. Of those, one in four has human epidermal growth factor receptor 2 positive (HER2 +) disease [2, 3], for whom consecutive lines of HER2-targeted therapy combined with chemotherapy or endocrine therapy are advised [4, 5]. Since 2013, the combination of pertuzumab, trastuzumab and taxane is the recommended first-line treatment in the Netherlands, based on the results of the CLEOPATRA trial (hazard ratio for overall survival with the addition of pertuzumab to trastuzumab and chemotherapy was 0.69, 95%*CI*:0.58–0.82) [6]. As second or subsequent lines of therapy, several agents in combination with HER2- targeted therapies are possible, such as capecitabine-trastuzumab-tucatinib, capecitabine-neratinib, T-DM1, or endocrine therapy in case of hormone receptor positive disease [5]. As of 2022, trastuzumab-deruxtecan (T-DXD) and a combination of capecitabine, trastuzumab and tucatinib have been added to the treatment landscape of patients with HER2 + advanced breast cancer (ABC) [5]. Both the French ESME and Dutch SONABRE real-world registries have shown an improvement in the overall survival of patients with HER2 + metastatic breast cancer (including both de novo and metachronous metastases) since the introduction of pertuzumab and T-DM1 [7, 8]. The time trends in progression-free and overall survival in patients with de novo HER2+ ABC in specific have not been reported so far. Furthermore, recent data on systemic and locoregional therapy use in patients with de novo HER2+ ABC in real-world are currently unknown.

Real world data (RWD) can provide real-world evidence on disease characteristics, treatment patterns and (long-term) outcomes. It can provide insight into the uptake and impact of new drugs and provide important mirror information to clinicians. RWD is time- and place-specific and limited by its observational design. It does however inform patient (advocates), physicians, industry, regulatory agents, and other stakeholders, and has gained recognition in the academic society [9, 10].

The main objective of this study was to determine whether the real-world first-line progression-free survival (PFS) of patients diagnosed with de novo HER2+ ABC has improved since the introduction of pertuzumab in 2013. We, therefore, compared the PFS of patients diagnosed in 2013–2017 with the PFS of patients diagnosed in 2008–2012. In addition, we aimed to determine differences between the two time periods in terms of overall survival (OS) and the use of systemic and locoregional therapies.

## Patients and methods

### SONABRE registry and patient population

The SOutheast Netherlands Advanced BREast cancer (SONABRE) Registry (NCT-03577197) is an ongoing prospectively maintained retrospective cohort study including all patients aged 18 years or older and diagnosed with metastatic breast cancer since 2007 in the Southeast of the Netherlands. Patient (age, comorbidities, WHO performance status) and tumour (clinical tumour and nodal stage of the primary tumour, hormone receptor subgroup, number and site of metastatic disease) characteristics, as well as treatment information (surgery, radiotherapy, and systemic treatment, neo-adjuvant, adjuvant and palliative), dates of progression, and date of death, were collected from medical files by trained registration clerks. The Medical Research Ethics Committee of Maastricht University Medical Centre approved the registry (15-4-239). For the present study, we selected all patients diagnosed with and systemically treated for de novo HER2+ ABC in 2008–2017 in ten hospitals, including one comprehensive cancer centre, six teaching and three non-teaching hospitals. The last follow-up was collected in 2021. Data lock was on September 3rd, 2021.

### Definitions

De novo metastatic disease was defined as the radiologic and/or pathologic diagnosis of at least one metastatic lesion at primary diagnosis or within three months thereafter. Receptor status was locally determined. HER2-positivity was defined as a positive in situ hybridization (ISH) result or an immunohistochemistry score of 3 + of a metastatic lesion, or if not available, of the primary tumour [11, 12]. Hormone receptor positivity was defined as nuclear staining of ≥ 10% for the oestrogen and/or progesterone receptor. Surgery of the primary tumour includes breast surgery with or without axillary surgery.

### Endpoints and statistical analyses

Patient and tumour characteristics were described per period of diagnosis and compared between the two time periods using Chi-square tests. Median follow-up time was determined per period using the reverse Kaplan–Meier method.

PFS was determined from the start of first-given systemic therapy for ABC until the reported progression of disease or death, whichever occurred first. Patients were censored at the start of a new line of therapy without progression or at the date of the last follow-up. OS was determined from the date of diagnosis until the date of death or censored at last follow-up date. PFS and OS were calculated using the Kaplan–Meier method per period. Multivariable Cox proportional hazards regression modelling was performed to compare the PFS and OS rates between the two periods. Potential confounding factors (including age, any comorbidity, cardiovascular comorbidity, hormone receptor status, initial metastatic site and number of organs involved) were included in the multivariable model if the p-value was < 0.05 in the univariable model. The OS by period of diagnosis was also studied in all patients diagnosed with de novo HER2 + ABC (i.e. including those not systemically treated) to prevent the risk of selection bias.

To identify patient subgroups with a different change in PFS or OS over time, we performed an explorative subgroup analyses by age, hormone receptor status and number of organs involved.

To present the use of systemic therapy, we reported first-given treatment regimens (HER2-targeted therapy, chemotherapy and/or endocrine therapy) and the use of systemic agents within the first five years from diagnosis (any HER2-targeted therapy, pertuzumab and T-DM1) per period using competing risk methodology [8]. Use of locoregional therapy (surgery of the primary tumour, and surgery and radiotherapy of a metastatic site) within the first year after diagnosis was determined per time period using competing risk methodology.

Explorative subgroup analyses were performed to determine differences in the effect of period of diagnosis on PFS and OS by age at diagnosis (< 65 versus 65 + years), hormone receptor status (HR + /HER2 + versus HR − /HER2 +) and the number of organs involved (single versus multiple) using likelihood-ratio tests (i.e. test for interaction). Furthermore, first-given systemic therapy and surgery of the primary tumour or a metastatic site by age, hormone receptor status and number of organs involved were determined by the period of diagnosis.

## Results

Among the 596 patients diagnosed with HER2+ ABC in 2008–2017, 179 (30%) patients were diagnosed with de novo HER2+ ABC (which was 32% (94/297) in 2013–2017 and 28% (85/301) in 2008–2012). Among those, 166 (93%) were systemically treated (*n* = 85 (95%) in 2013–2017 and *n* = 81 (90%) in 2008–2012). Baseline characteristics were similar in 2013–2017 versus 2008–2012: median age was 55 versus 55 years, 69% versus 65% of patients had hormone receptor positive disease, 66% versus 69% of patients had visceral metastases and 7% versus 7% central nervous system metastases (Table 1). Distant metastases were detected during the diagnostic work-up of the primary tumour (i.e. screening) in, respectively, 79% and 83% of patients. Clinical tumour and nodal stages were also comparable. Of note, there was a statistically non-significant trend towards a lower proportion of patients with cardiovascular comorbidities (18% versus 30%) and a higher frequency of soft tissue metastases (47% versus 36%) in 2013–2017 versus 2008–2012. Median follow-up time was 66.9 months (95%*CI*: 52.0–81.7) for the period 2013–2017 and 127.7 months (95% confidence interval (*CI*): 121.4–134.0) for the period 2008–2012, during which, respectively, 44 (52%) and 72 (89%) patients had died.

**Table 1:** Baseline characteristics of patients systemically treated for de novo HER2-positive advanced breast cancer in 2008–2012 and 2013–2017

		Period (year of ABC diagnosis)		
Characteristics		2008–2012 *N* = 81	2013–2017 *N* = 85	*P-*value
		*N *(%)	*N *(%)	
Age at diagnosis ABC				0.47
< 65 years		60 (74)	67 (79)	
≥ 65 years		21 (26)	18 (21)	
Median (IQR)		55 (46–67)	55 (47–63)	0.90
Comorbidity				
Any		51 (63)	55 (65)	0.82
Cardiovascular		24 (30)	15 (18)	0.07
WHO performance score				0.71
WHO 0–1		43 (88)	71 (90)	
WHO ≥ 2		6 (12)	8 (10)	
* Missing*		*32*	*6*	
Clinical T-stage primary tumour				0.46
cT1		15 (19)	13 (15)	
cT2		24 (31)	35 (41)	
cT3		10 (13)	13 (15)	
cT4		29 (37)	24 (28)	
* cTx*		*3*	*0*	
Clinical N-stage primary tumour				0.48
cN0		10 (13)	14 (17)	
cN1		42 (56)	38 (46)	
cN2-3		23 (31)	30 (37)	
* cNx*		*6*	*3*	
Mode of detection metastatic disease				0.53
Screen-detected		67 (83)	67 (79)	
Symptomatic		14 (17)	18 (21)	
Hormone receptor status^§^				0.58
Positive		53 (65)	59 (69)	
Negative		28 (35)	26 (31)	
Number of organs involved				0.42
Single organ		35 (43)	42 (49)	
Multiple organs		46 (57)	43 (51)	
Initial metastatic sites^a^				
Bone		56 (69)	51 (60)	0.22
* Bone only*		19 (24)	17 (20)	0.64
Soft tissue^1^		29 (36)	40 (47)	0.14
Visceral^2^		56 (69)	56 (66)	0.66
* Lung*		27 (33)	21 (25)	0.22
* Liver*		41 (51)	40 (47)	0.65
* Pleura*		8 (10)	3 (4)	0.10
CNS^3^		6 (7)	6 (7)	0.93

Percentages may not add up to 100% because of rounding

*ABC* advanced breast cancer, *CNS* central nervous system, *HER*2 Human Epidermal growth factor Receptor 2, *HR *hormone receptor, *IQR* inter quartile range, *N*  nodal, *T*  tumour, *WHO* World Health Organization

^§^Receptor status of the metastatic site (25% in 2008–2012 versus 32% in 2013–2017) or, if not available, of the primary tumour (75% versus 68%)

^a^Sum of percentages exceeds 100 because multiple options are possible

^1^Lymph nodes, skin and eye

^2^Liver, lung, pleura, peritoneum, gastrointestinal track, kidney, adrenal and ovaries

^3^Brain and leptomeningeal

Italics is used to highlight the unknowns(*cTx*, *cNx*)/missings, and to provide subheading. -‘Bone only’ is part of Bone- ‘Lung, Liver, Pleura’ is part of Visceral

### Outcomes

**Fig. 1:**
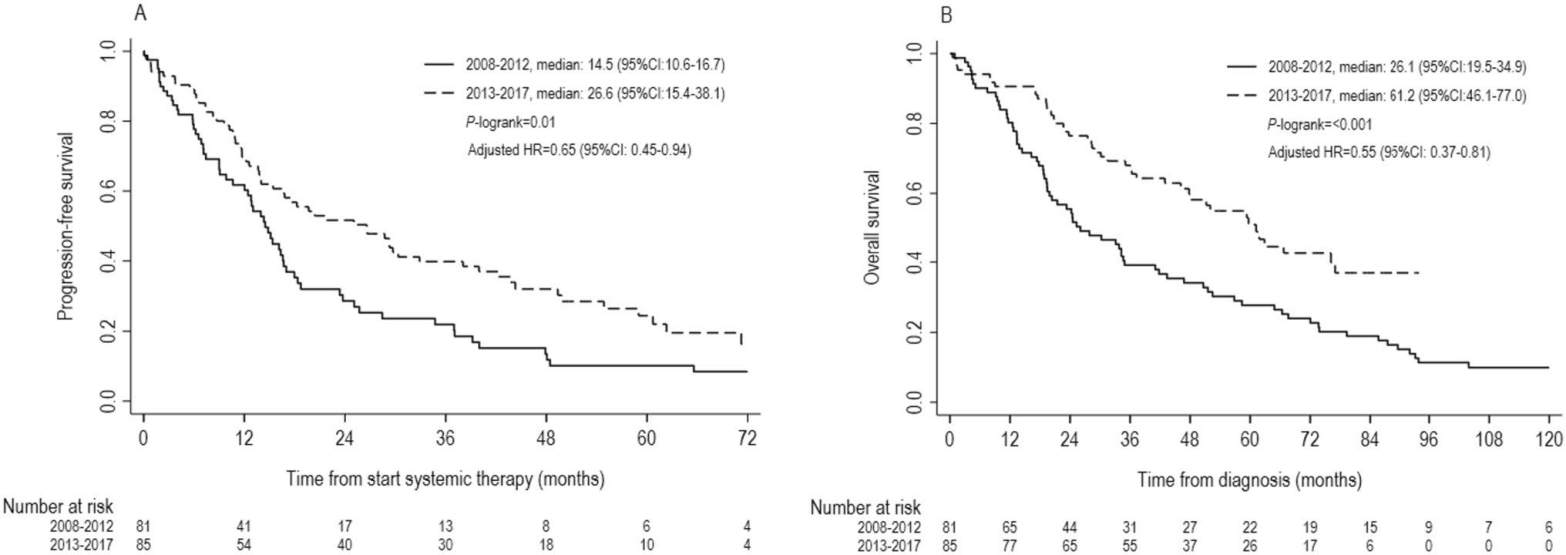
First-line progression-free survival (**A**) and overall survival (**B**) in patients systemically treated for de novo HER2-positive advanced breast cancer in 2013–2017 versus 2008–2012

Median first-line PFS was 26.6 months (95%*CI*: 15.4–38.1) for patients diagnosed in 2013–2017 and 14.5 months (95%*CI*: 10.6–16.7) for patients diagnosed in 2008–2012 (Fig. 1A). Five-years PFS rates were 24% (95%*CI*: 15%-35%) for patients diagnosed in 2013–2017 and 10% (95%*CI*: 4%-19%) for patients diagnosed in 2008–2012.

Median OS was 61.2 months (95%*CI*: 46.1–77.0) for patients diagnosed in 2013–2017 versus 26.1 months (95%*CI*: 19.5–34.9) in 2008–2012 (Fig. 1B). Five-year OS rates were 51% (95%*CI*: 39%-62%) for patients diagnosed in 2013–2017 versus 28% (95%*CI*: 18%-38%) in 2008–2012.

PFS and OS were longer in patients diagnosed in 2013–2017 when compared with 2008–2012 (PFS: adjusted hazard rate ratio (HR) = 0.65, 95%*CI*: 0.45–0.94, *p* = 0.02, OS: adjusted *HR* = 0.55, 95%*CI*: 0.37–0.81, *p* = 0.002, Table 2).

When including the 13 patients not systemically treated (sensitivity analysis), the median and five-year OS in all patients diagnosed with de novo HER2+ ABC were 52.0 months (95%*CI*: 36.1–66.7) and 46% (95%*CI*: 35%-57%) in 2013–2017 and 25.3 months (95%*CI*: 19.3–34.7) and 28% (95%*CI*: 19%-37%) in 2008–2012 (adjusted *HR* = 0.64, 95%*CI*: 0.44–0.93, *p* = 0.02; data not further shown).

**Table 2:** Univariable and multivariable analyses for progression-free and overall survival in patients with de novo HER2+ advanced breast cancer

	Progression-free survival (*N* = 166, events = 123)				Overall survival (*N* = 166, events = 116)			
	Univariable analysis		Multivariable analysis		Univariable analysis		Multivariable analysis	
	Hazard ratio (95% *CI*)	*P-value*	Hazard ratio (95% *CI*)	*P-value*	Hazard ratio (95% *CI*)	*P-value*	Hazard ratio (95% *CI*)	*P-value*
Incidence period								
2008–2012	Ref.		Ref.		Ref.		Ref.	
2013–2017	0.63 (0.44–0.90)	0.011	0.65 (0.45–0.94)	0.022	0.51 (0.35–0.75)	0.001	0.55 (0.37–0.81)	0.002
Age								
< 65 years	Ref.		Ref.		Ref.		Ref.	
≥ 65 years	2.92 (1.96–4.36)	< 0.001	2.74 (1.84–4.09)	< 0.001	3.05 (2.03–4.59)	< 0.001	3.29 (2.07–5.24)	< 0.001
Comorbidity								
No	Ref.		NA		Ref.		NA	
Yes	1.06 (0.73–1.53)	0.73			1.27 (0.88–1.86)	0.21		
Cardiovascular disease								
No	Ref.		NA		Ref.		Ref.	0.53
Yes	1.29 (0.86–1.93)	0.22			1.56 (1.03–2.34)	0.03	1.01 (0.64–1.60)	
Hormone receptor								
Positive	Ref.		NA		Ref.		NA	
Negative	0.89 (0.61–1.30)	0.54			1.08 (0.73–1.60)	0.68		
Initial metastatic sites								
Bone only	Ref.		NA		Ref.		Ref.	
Soft tissue^1^ without visceral or CNS	1.06 (0.54–2.09)	0.86			0.74 (0.33–1.65)	0.47	0.76 (0.32–1.81)	0.53
Visceral^2^ without CNS	1.05 (0.68–1.61)	0.84			1.17 (0.74–1.84)	0.69	1.05 (0.58–1.90)	0.87
CNS^3^	1.55 (0.70–3.43)	0.28			2.22 (1.09–4.53)	0.03	2.07 (0.85–5.05)	0.11
Number of organs involved								
Single organ	Ref.		Ref.		Ref.		Ref.	
Multiple organs	1.39 (0.97–1.99)	0.075	1.38 (0.96–1.98)	0.080	1.63 (1.12–2.36)	0.01	1.58 (0.97–2.58)	0.07

*CNS* central nervous system, *NA* not analysed (not included because of a P-value > 0.20 in the univariable analysis)

^1^Lymph nodes, skin and eye

^2^Liver, lung, pleura, peritoneum, gastrointestinal track, kidney, adrenal and ovaries

^3^Brain and leptomeningeal

In the explorative subgroup analyses, the relative improvement in PFS in 2013–2017 versus 2008–2012 was numerically larger in patients aged under 65 years at diagnosis (*HR* = 0.61) when compared with patients aged 65 + years (*HR* = 0.79), in patients with HR−/HER2+ (*HR* = 0.39) versus HR + /HER2 + disease (HR = 0.82) and in patients with multiple organs involved (*HR* = 0.48) versus a single metastatic site (*HR* = 0.80), although the tests for interaction did not reach statistical significance (Appendix Table 4 and Appendix Fig. 4). The subgroup analyses for OS results were in line with the PFS results (Appendix Table 4 and Appendix Fig. 5).

**Fig. 2:**
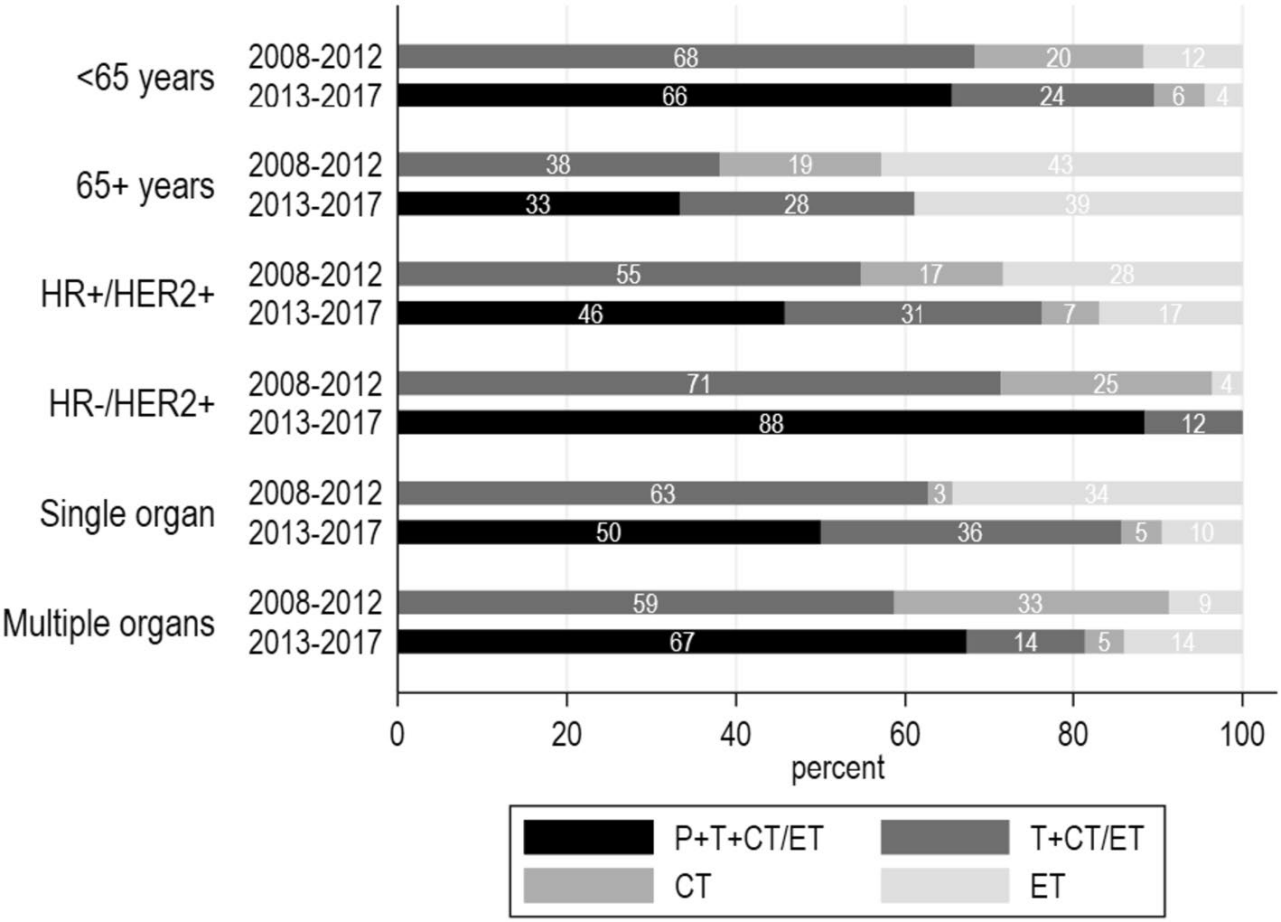
First-given systemic treatment in 2008–2012 and 2013–2017 by age at diagnosis, hormone receptor status and number of organs involved. *ET* endocrine therapy, *CT* chemotherapy, *CT*/*ET* chemotherapy or endocrine therapy, *P* pertuzumab, *T* trastuzumab

### Systemic therapy

The use of HER2-targeted therapy as part of first-given systemic therapy was higher in patients diagnosed in 2013–2017 (84%) when compared with 2008–2012 (60%, *p* = 0.001), whereas the use of chemotherapy alone and endocrine therapy alone decreased (Table 3). This trend was seen in all subgroups of patients, with the exception of a constant use of endocrine monotherapy in patients aged 65 + years (i.e. 39% in 2013–2017 versus 43% in 2008–2012) and in patients with metastases in multiple organs (i.e. 14% versus 9%) (Fig. 2). First-given systemic therapy comprised pertuzumab in 59% of patients diagnosed in 2013–2017 versus 0% in 2008–2012 (Table 3). In 2013–2017, pertuzumab use as first-given therapy varied between 33% in patients aged 65 + years and 88% for patients with HR−/HER2 + disease (Fig. 2).

**Table 3:** First-given systemic therapy in patients systemically treated for de novo HER2-positive advanced breast cancer in 2008–2012 and 2013–2017

		Period (year of ABC diagnosis)	
		2008–2012 *N* = 81	2013–2017 *N* = 85
		*N (%)*	*N (%)*
HER2-targeted therapy		49 (60)	71 (84)
Chemotherapy plus HER2-targeted therapy		41 (51)	63 (74)
*Pertuzumab plus trastuzumab*		*0*	*49* (*58*)
*Trastuzumab*		41 (51)	14 (16)
Endocrine therapy plus trastuzumab		6 (7)	6 (7)
HER2-targeted therapy alone		2 (3)	2 (2)
*Pertuzumab plus trastuzumab*		*0*	*1* (*1*)
*Trastuzumab*		2 (3)	1 (1)
Chemotherapy without HER2-targeted therapy		16 (20)	4 (5)
Endocrine therapy without HER2-targeted therapy		16 (20)	10 (12)

**Fig. 3:**
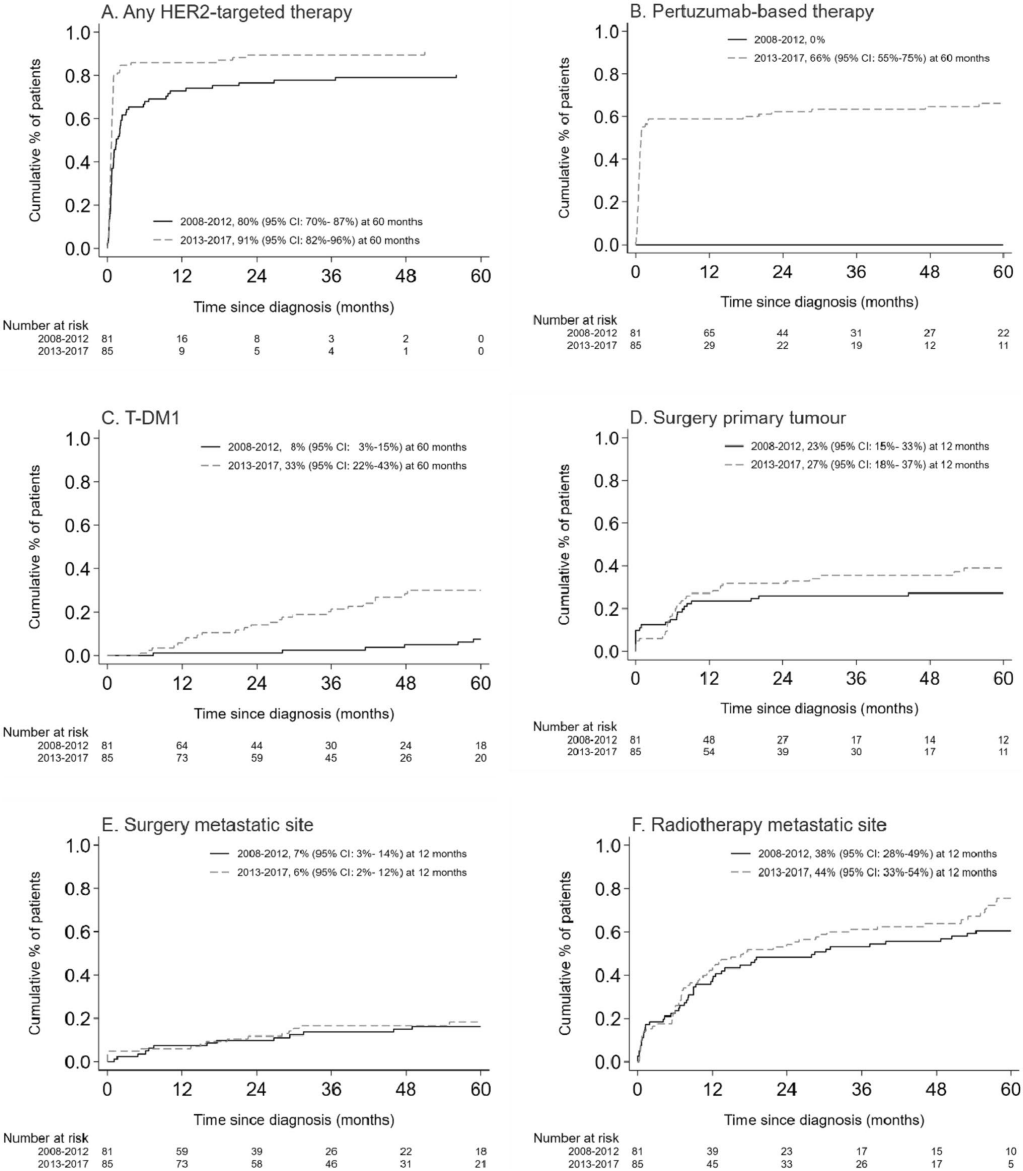
Time to first use of HER2-targeted therapy (**A** any HER2-targeted therapy, **B** pertuzumab and **C** T-DM1) and use of locoregional therapy (**D** surgery of the primary tumour, **E** surgery of a metastatic site, and **F** radiotherapy of a metastatic site) in 2013–2017 versus 2008–2012 in patients diagnosed with de novo HER2 + advanced breast cancer within the real-world SONABRE Registry.* ‘*Number at risk’ comprises the number of patients alive who did not receive the treatment of interest before, these patients are theoretically still eligible (‘at risk’) to undergo the treatment of interest

The use of any type of HER2-targeted therapy during the first five years of follow-up was slightly higher in 2013–2017 than in 2008–2012 (i.e. respectively 91% and 80%, Fig. 3A). The use of pertuzumab-based therapy and T-DM1 were, respectively, 66% and 33% in 2013–2017 and 0% and 8% in 2008–2012 at 60 months (Figs. 3B, C). Pertuzumab was mainly used as first-given systemic therapy (Fig. 3B).

During the follow-up period 483 lines of systemic therapy were observed. Of these, 30 (6%) were given as part of a clinical trial and 349 included HER2-targeted therapy. These lines of HER2-targeted therapy included trastuzumab without pertuzumab (*n* = 237), trastuzumab plus pertuzumab (*n* = 64), T-DM1 (*n* = 39), lapatinib (*n *= 8), or neratinib (*n* = 1).

### Locoregional therapy

The use of locoregional therapy did not differ between the periods: in 2013–2017 and 2008–2012, respectively, 27% and 23% of patients underwent surgery of the primary tumour, 6% and 7% surgery of a metastatic site and 44% and 38% radiotherapy of a metastatic site during the first year following diagnosis (Figs. 3D–F). Surgery of the primary tumour was mainly performed within the first year after diagnosis, whereas surgery or radiotherapy of a metastatic site was performed throughout the entire disease course.

Surgery of the primary tumour was performed more often in patients aged < 65 years and in patients with single organ involvement, but the use did not differ between periods within patient subgroups (Appendix Fig. 6). Surgery of a metastatic site did not differ between subgroups, except for a low use in patients aged 65 + years, and was constant over time (Appendix Fig. 7).

## Discussion

The prognosis of patients diagnosed with de novo HER2+ ABC in the Southeast of the Netherlands has improved considerably since 2013. One in four patients diagnosed in 2013–2017 were alive and free from progression on first-given therapy five years after start of treatment of de novo HER2+ ABC, and more than half of the patients were alive at five years. This advancement in prognosis is likely to be largely explained by the changes in systemic therapy, as baseline characteristics and use of locoregional therapy remained mostly the same.

The observed improvement in PFS in our study (*HR* = 0.65 (95% *CI*: 0.45–0.94), where 59% of patients received pertuzumab in 2013–2017, was similar to the results of the CLEOPATRA trial (*HR* = 0.69, 95% *CI*:0.58–0.81), where 100% received pertuzumab in the intervention arm [13]. The higher improvement observed in our real-world study could be attributable to an increased use of HER2-targeted therapy in general (i.e. 84% in 2013–2017 versus 60% in 2008–2012) and/or a higher efficacy of pertuzumab in patients with de novo HER2+ ABC. The relative improvement in PFS is expected to be larger in patients diagnosed with de novo than metachronous HER2+ ABC, since pertuzumab and trastuzumab are more effective in chemo- and HER2-targeted therapy-naïve patients [14–16]. In the CLEOPATRA trial, for example, 47% of patients had received (neo)adjuvant chemotherapy and 11% (neo)adjuvant trastuzumab [13]. Moreover, differences in clinical, pathological and molecular characteristics between patients with de novo and metachronous metastases may also play a role in differences in treatment efficacy [17]. We, therefore, recommend researchers to incorporate ‘de novo versus metachronous ABC’ as stratification factor when designing and analysing clinical trials.

Besides changes in HER2-targeted therapy, the improvement in PFS may also be partly explained by other changes in breast cancer care. A different selection of patients who started systemic therapy may have played a role. Earlier, anti-HER2 treatment with endocrine therapy might have been used as a non-toxic first-given therapy in elderly patients, whereas nowadays chemotherapy combined with anti-HER2 treatment is standard first-given therapy. Indeed, the proportion of patients systemically treated was slightly lower in 2013–2017 (90%) as in 2008–2012 (95%), but OS advancements were still significantly improved in the total patient population (*HR* = 0.64). Baseline characteristics were similar in the two time periods, except for a lower proportion of patients with cardiovascular disease in 2013–2017 (18%) compared with 2008–2012 (30%). This may also partly explain the lower use of HER2-targeted therapy in 2008–2012 and account for part of the PFS difference observed in this study. Furthermore, a higher proportion of patients had soft tissue metastases (mainly distant lymph node metastases) in 2013–2017 as compared with 2008–2012. The classification of contralateral lymph nodes as regional recurrence or distant metastases has been discussed for years. In 2014, a Delphi consensus was published defining contralateral lymph nodes as distant metastases [18]. This consensus may have led to the inclusion of more patients with contralateral lymph nodes. The presence of soft tissue metastases was, however, not an independent prognostic factor in our PFS analyses, suggesting a small impact on the PFS improvement, if any. An improved PFS in more early years due to stage migration or a more early diagnosis is expected to be small or even absent since the mode of detection and clinical tumour and nodal stage were similar in both periods. In the Netherlands, dissemination in terms of distant staging was only indicated in patients with stage III or IV breast cancer. Since 2013, distant staging was also recommended in patients with stage IIB and more often with PET-CT scanning [12, 19]. In addition, advancements in (full-field) digital mammography and the use and improved quality of breast MRI in the diagnostic process may have resulted in an upstaging of tumour size [20]. These changes did, however, not lead to more advanced primary breast cancers in our study population nor to a higher rate of screen-detected de novo breast cancer metastases. We, therefore, expect that the impact of these changes in screening and imaging had only a marginal, if any, effect on the observed PFS improvement.

To our knowledge, we are the first to report real-world PFS outcomes in the pertuzumab time era for patients with de novo HER2 + ABC in specific, showing that one in four patients were alive and free of progression five years after the start of systemic treatment. In the trastuzumab era (2008–2012), five-year PFS was 10%, similar to previous cohorts [21, 22]. Lambertini et al. observed a five-year PFS of around 15% in patients with de novo HER2+ ABC treated with first-line trastuzumab in Italy in 2000–2013 [21]. Wong et al. reported the outcomes for patients reaching radiological complete response (13%) or not (87%) in patients with de novo HER2 + ABC treated with first-line trastuzumab in 1998–2015 in Yale or MD Anderson Cancer Centres [22]. Twenty percent of these patients received pertuzumab in addition to trastuzumab-based treatment. Five-year PFS rates were 100% for patients with a radiological complete response and 12% for patients who did not.

In our real-world study, the ten-year OS rate of patients with de novo HER2+ ABC in 2008–2012 was 10% (Fig. 1B). In line, the ten-year OS was approximately 10% in patients diagnosed with de novo HER2+ ABC in 2001–2009 in Canada [23]. In France, ten-year OS was around 20% for patients diagnosed with de novo or metachronous HER2+ ABC in 2008–2010 [7]. This higher rate could partly be explained by the exposure to pertuzumab (5%) and T-DM1 (15%) in this period. OS is generally longer in patients included in clinical trials than in real-world. Long-term follow-up of 40 women who received chemotherapy plus trastuzumab as part of the phase II UPCI 99–058 trial showed eight- and ten-year OS rates of 22.5% and 12.5% [24]. Patients included in the chemotherapy plus trastuzumab arm of the CLEOPATRA trial had an eight-year OS of 23% (ten-year OS not available) [6]. Patients included in the pertuzumab arm achieved an eight-year OS rate of 37%. These high long-term survival rates suggest that long-term control or even cure is possible for a subpopulation of HER2 + ABC, and shows the need to define the optimal duration of HER2-targeted maintenance therapy [25].

Our study showed that patient age was the sole independent prognostic factor for OS (Table 2). Other patient and tumour characteristics were not statistically significantly associated with OS. Two American real-world studies examining potential prognostic factors for survival in patients treated with trastuzumab for de novo HER2+ ABC found mixed results [22, 26]. This requires further study.

In patients with de novo ABC, the role of breast surgery is under debate. Observational studies have shown that locoregional treatment of the primary breast tumour might improve survival in a subgroup of patients with de novo disease, although clinical trials were inconclusive [27–30]. In addition, surgery and radiotherapy of a metastatic site are considered for symptom control or treatment aiming at long-term remission in the case of oligometastatic disease [9]. As the prognosis of patients with de novo HER2+ ABC is improving, locoregional therapy may become increasingly important and should be subject to future research. Of note, in our study, one in four patients with de novo HER2+ ABC received primary locoregional breast therapy, which did not differ between the two time periods [22]. This was consistent with the 22% reported in de novo HER2 + ABC patients in the French ESME cohort in 2008–2014, but lower than the 48% reported in the Italian GIM cohort in 2000–2013 [2, 21].

The strengths of this study are the real-world setting and the long follow-up period providing five-year survival rates and the use of therapies for patients diagnosed with de novo HER2+ ABC. Although we included all patients diagnosed with de novo HER2+ ABC in ten hospitals in a ten-year period, the low number of patients limited us in studying subgroups. Our subgroup analyses were, therefore, of explorative character and should be confirmed in larger populations. We observed a higher PFS and OS improvement over time in patients with HR-/HER2 + than in patients with HR + /HER2+ ABC. This is expected to be associated with a higher use of HER2-targeted therapy and pertuzumab in patients with HR-/HER2+ ABC, as high-level evidence on a differential efficacy of HER2-targeted therapy by hormone receptor status is lacking. In this observational study, we cannot determine the individual contribution of changes in systemic therapy to the PFS and OS improvements. Nevertheless, it provides recent changes in clinical practice and prognosis. Other limitations are inherent to the observational nature of this study, using physicians’ notes for the data collection, resulting in some missing information on baseline characteristics. In addition, the epidemiological data reported in this regional study are time- and place-specific, generally limiting the generalizability of the results to other hospitals and countries. However, the resemblance with data reported for the pre-pertuzumab era supports generalizability of our study results for the pertuzumab era. Including different hospital types is considered a strength of this study. Nowadays, the PFS and OS are expected to be longer as reported in this study since an underuse of pertuzumab was observed in 2013 and 2014, whereas pertuzumab use was stable in 2015–2017 [8]. The OS is also expected to further increase due to new HER2-targeted treatment options [5].

## Conclusions

In the southeast of the Netherlands, PFS and OS improved significantly for patients diagnosed with de novo HER2 + ABC since 2013, probably mainly related to changes in HER2-targeted therapy. Nowadays, one in two patients are alive and one in four alive and free of progression five years after diagnosis. Locoregional therapies may become of added value given these improved long-term outcomes. The prognosis is expected to be even better for currently diagnosed patients due to the availability of new HER2-targeted therapies.

## Figures and Tables

**Fig. 4:**
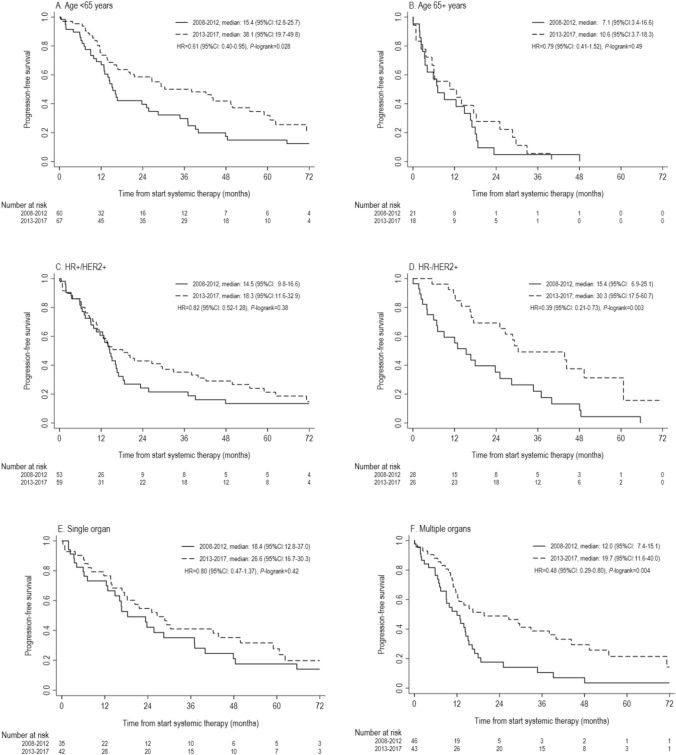
Progression-free survival in patients systemically treated for de novo HER2 + advanced breast cancer in 2013–2017 versus 2008–2012; for **A** patients aged < 65 vs. **B** 65 + years ( P -value interaction = 0.45), **C** hormone receptor positive vs. **D** hormone receptor negative disease ( P -value interaction = 0.085); and **E** single organ vs. **F** multiple organ metastases ( P -value interaction = 0.16)

**Fig. 5:**
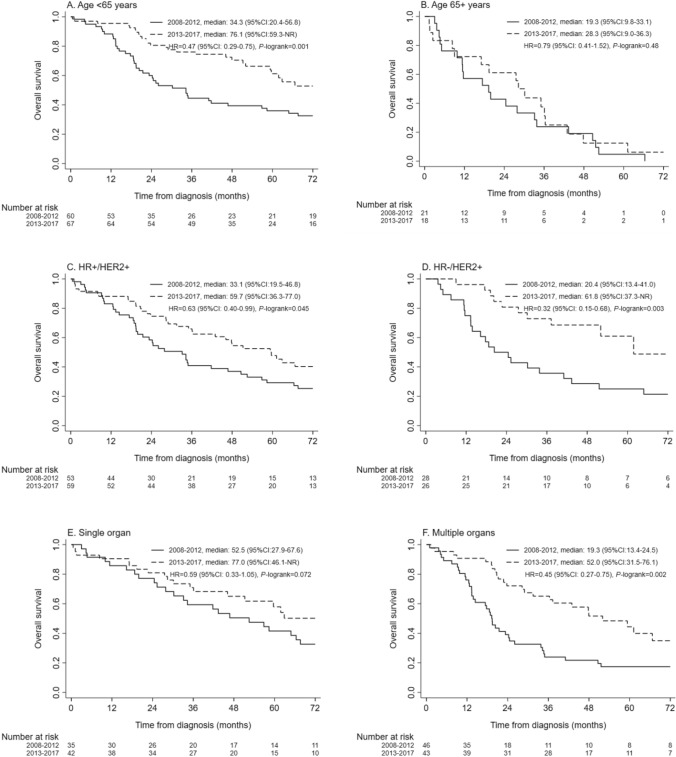
Overall survival in patients systemically treated for de novo HER2 + advanced breast cancer in 2013–2017 versus 2008–2012; for **A** patients aged < 65 vs. **B** 65 + years, **C** hormone receptor positive vs. **D** hormone receptor negative disease; and **E** single organ vs. **F** multiple organ metastases

**Fig. 6:**
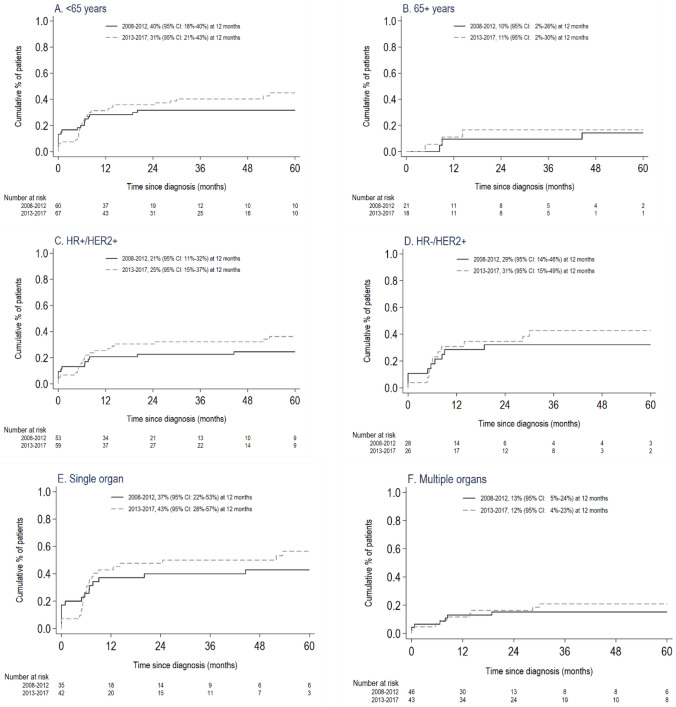
Time to surgery of the primary tumour in patients systemically treated for de novo HER2 + advanced breast cancer in 2013–2017 versus 2008–2012 in patients **A** aged < 65 years, **B** aged 65 + years, **C** with HR + /HER2- disease, **D** with HR-/HER2 + disease, **E** with a single metastatic site, and **F** with metastases in multiple organs

**Fig. 7:**
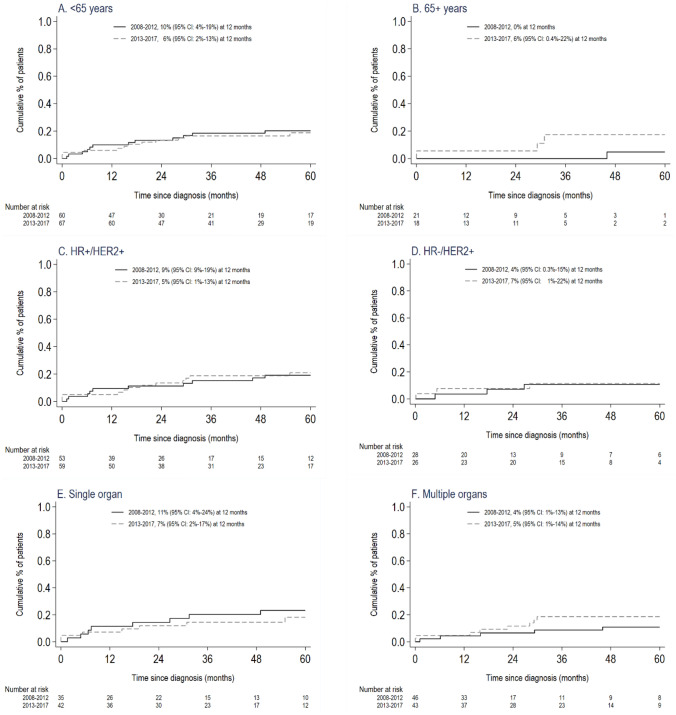
Time to surgery of a metastatic site in patients systemically treated for de novo HER2+ advanced breast cancer in 2013-2017 versus 2008-2012 in patients **A** aged <65 years, **B** aged 65+ years, **C** with HR+/HER2- disease, **D** with HR-/HER2+ disease, **E** with a single metastatic site, and **F** with metastases in multiple organs

## Data Availability

The datasets generated during and/or analysed during the current study are not publicly available but are available from the corresponding author on reasonable request.

## Appendix

See Table 4

**Table 4:** Explorative subgroup analyses for the association between period and progression-free and overall survival in patients with de novo HER2 + advanced breast cancer by age at diagnosis, hormone receptor status and metastatic spread

Progression-free survival	Patients (n)/events (n)		Median (95% CI) (in months)		Univariable regression		Test for interaction
	2008–2012	2013–2017	2008–2012	2013–2017	HR (95% CI)	P-value	P-value
All patients	81/63	85/60	14.5 (10.6–16.7)	26.6 (15.4–38.1)	0.63 (0.44–0.90)	0.011	
Age at diagnosis ABC							0.45
< 65 years	60/42	67/42	15.4 (12.8–25.7)	38.1 (19.7–49.8)	0.61 (0.40–0.95)	0.028	
≥ 65 years	21/21	18/18	7.1 (3.4–16.6)	10.6 (3.7–18.3)	0.79 (0.41–1.52)	0.49	
Hormone receptor status							0.085
Positive	53/38	59/43	14.5 (9.8–16.6)	18.3 (11.6–32.9)	0.82 (0.52–1.28)	0.38	
Negative	28/25	26/17	15.4 (6.9–25.1)	30.3 (17.5–60.7)	0.39 (0.21–0.73)	0.003	
Number of organs involved							0.16
Single organ	35/28	42/29	18.4 (12.8–37.0)	26.6 (16.7–30.3)	0.80 (0.47–1.37)	0.42	
Multiple organs	46/35	43/31	12.0 (7.4–15.1)	19.7 (11.6–40.0)	0.48 (0.29–0.80)	0.004	
**Overall survival**							
All patients	81/72	85/44	26.1 (19.5–34.9)	61.2 (46.1–77.0)	0.51 (0.35–0.75)	0.001	
Age at diagnosis ABC							0.30
< 65 years	60/51	67/28	34.3 (20.4–56.8)	76.1 (59.3-NR)	0.47 (0.29–0.75)	0.001	
≥ 65 years	21/21	18/16	19.3 (9.8–33.1)	28.3 ( 9.0–36.3)	0.79 (0.41–1.52)	0.48	
Hormone receptor status							0.097
Positive	53/45	59/34	33.1 (19.5–46.8)	59.7 (36.3–77.0)	0.63 (0.40–0.99)	0.045	
Negative	28/27	26/10	20.4 (13.4–41.0)	61.8 (37.3-NR)	0.32 (0.15–0.68)	0.003	
Number of organs involved							0.54
Single organ	35/29	42/19	52.5 (27.9–67.6)	77.0 (46.1-NR)	0.59 (0.33–1.05)	0.072	
Multiple organs	46/43	43/25	19.3 (13.4–24.5)	52.0 (31.5–76.1)	0.45 (0.27–0.75)	0.002	

See Appendix Figure 4 and 5 for the Kaplan–Meier curves

*ABC* advanced breast cancer, *CI* Confidence interval, *HR * Hazard rate Ratio, *NR* not reached, *OS* overall survival

See Figs. 4, 5, 6, 7
